# Dietary yeast-derived mannan oligosaccharides have immune-modulatory properties but do not improve high fat diet-induced obesity and glucose intolerance

**DOI:** 10.1371/journal.pone.0196165

**Published:** 2018-05-03

**Authors:** Lisa R. Hoving, Hendrik J. P. van der Zande, Amanda Pronk, Bruno Guigas, Ko Willems van Dijk, Vanessa van Harmelen

**Affiliations:** 1 Department of Human Genetics, Leiden University Medical Center, Leiden, The Netherlands; 2 Einthoven Laboratory for Experimental Vascular Medicine, Leiden University Medical Center, Leiden, The Netherlands; 3 Department of Parasitology, Leiden University Medical Center, Leiden, The Netherlands; 4 Department of Molecular Cell Biology, Leiden University Medical Center, Leiden, The Netherlands; 5 Department of Medicine, division Endocrinology, Leiden University Medical Center, Leiden, The Netherlands; Oklahoma State University, UNITED STATES

## Abstract

The indigestible mannan oligosaccharides (MOS) derived from the outer cell wall of yeast *Saccharomyces cerevisiae* have shown potential to reduce inflammation. Since inflammation is one of the underlying mechanisms involved in the development of obesity-associated metabolic dysfunctions, we aimed to determine the effect of dietary supplementation with MOS on inflammation and metabolic homeostasis in lean and diet-induced obese mice. Male C57BL/6 mice were fed either a low fat diet (LFD) or a high fat diet (HFD) with, respectively, 10% or 45% energy derived from lard fat, with or without 1% MOS for 17 weeks. Body weight and composition were measured throughout the study. After 12 weeks of intervention, whole-body glucose tolerance was assessed and in week 17 immune cell composition was determined in mesenteric white adipose tissue (mWAT) and liver by flow cytometry and RT-qPCR. In LFD-fed mice, MOS supplementation induced a significant increase in the abundance of macrophages and eosinophils in mWAT. A similar trend was observed in hepatic macrophages. Although HFD feeding induced a classical shift from the anti-inflammatory M2-like macrophages towards the pro-inflammatory M1-like macrophages in both mWAT and liver from control mice, MOS supplementation had no effect on this obesity-driven immune response. Finally, MOS supplementation did not improve whole-body glucose homeostasis in both lean and obese mice.Altogether, our data showed that MOS had extra-intestinal immune modulatory properties in mWAT and liver. However these effects were not substantial enough to significantly ameliorate HFD-induced glucose intolerance or inflammation.

## Introduction

Obesity is associated with chronic low-grade inflammation. Obesity induces a phenotypic switch in the expanding white adipose tissue (WAT) from an anti-inflammatory towards a pro-inflammatory state which is characterized by an increase in M1-like macrophages, cytotoxic T cells, B cells, and neutrophils, whereas the numbers of M2-like macrophages, regulatory T cells, and eosinophils are reduced [1–5].

WAT inflammation results in the release of pro-inflammatory cytokines and fatty acids in the circulation, which are key mediators in inducing insulin resistance and inflammation in other organs, including the liver [6]. Inflammation in the insulin resistant liver is mainly characterized by high numbers of hepatic pro-inflammatory macrophages [7]. Obesity-associated inflammation is thought to eventually lead to the development of type 2 diabetes [8].

Dietary supplementation with mannan-oligosaccharides (MOS) has been suggested to modulate inflammation [9,10]. MOS are derived from the outer cell-wall membrane of bacteria, plants, or yeast and have been shown to be resistant to hydrolysis by the action of digestive enzymes in the human gut [11]. They are widely used in the animal industry as food supplements to reduce pathogenic contamination and to improve economic performance [12,13].

MOS supplementation was reported to lower the ileal gene expression of pro-inflammatory cytokines while increasing anti-inflammatory cytokines after challenging broilers with *Escherichia coli* [14]. Interestingly, there are also indications that MOS have extra-intestinal immune modulatory properties. Indeed, alveolar macrophages from pigs fed a MOS diet for two weeks showed reduced secretion of the pro-inflammatory cytokine *TNF-α* and increased secretion of the anti-inflammatory cytokine *IL-10* in response to *ex vivo* stimulation by lipopolysaccharide (LPS) [15]. In addition, MOS improved immune responses and growth efficiency of nursery pigs after experimental respiratory virus infection [16].

Since inflammation is one of the underlying mechanisms involved in the development of obesity-associated dysfunctions, we hypothesized that dietary MOS have extra-intestinal immune modulating properties and reduce inflammation in WAT and liver of obese mice. Therefore, we aimed to determine the effect of dietary supplementation with *saccharomyces cerevisiae*-derived MOS on inflammation in metabolic tissues and whole-body glucose tolerance in both lean and HFD-induced obese mice.

Altogether, we report that MOS supplementation slightly altered the immune cell composition of mWAT and liver in lean mice, but did not ameliorate HFD-induced glucose intolerance or inflammation.

## Materials and methods

### Mice and diet

Male C57BL/6J mice were purchased from Charles River (Maastricht, The Netherlands) and housed under temperature- and humidity-controlled conditions with a 12:12h light-dark cycle and free access to food and water. At the start of the experiment mice were 10 weeks of age. Mice (n = 10 per group) were fed a LFD or HFD (10% or 45% kcal derived from lard fat, respectively; D12450B and D12451, Research Diet Services, Wijk bij Duurstede, The Netherlands) supplemented with 1% MOS (LFD-M and HFD-M) or without (LFD and HFD). The rationale behind the usage of 1% MOS was based on a study performed in C57BL/6 mice, where addition of 1% MOS to the diet led to decreased fat accumulation in adipose tissue and liver [17]. MOS used in this study was derived from the outer cell wall of yeast *saccharomyces cerevisiae* (Actigen^®^, Alltech, Ridderkerk, Netherlands). After 17 weeks, mice were sedated, perfused with ice-cold PBS through the heart, and mesenteric subcutaneous white adipose tissue (mWAT), liver, as well as thymus and spleen were dissected for further analysis. Mouse experiments were performed in accordance with the Institute for Laboratory Animal Research Guide for the Care and Use of Laboratory Animals and had received approval from the University Ethical Review Board (Leiden University Medical Center, The Netherlands; permit no. 131031).

### Body weight, food intake, and body composition

During the diet intervention, body weight and food intake were measured weekly. Lean and fat mass were monitored every 4 weeks up to 12 weeks by using an EchoMRI-100 analyzer (Echo MRI, TX, USA).

### Stromal vascular cell isolation from mesenteric white adipose tissue

mWAT was dissected, rinsed in PBS and minced. Stromal vascular fraction (SVF) cells from mWAT were isolated as described previously [18]. Briefly, tissues were digested in a collagenase mixture (0.5 g/L collagenase [Type 1] in DMEM/F12 [pH 7.4] with 20 g/L of dialyzed bovine serum albumin [BSA, fraction V; Sigma, St Louis, USA]) for 1 h at 37°C, and filtered through a 236-μm nylon mesh. Upon centrifugation of the suspension (10 min, 200 g), the pelleted SVF was treated with red blood cell lysis buffer (BD Biosciences, CA, USA), stained with Aqua fixable live/dead stain (Invitrogen, Carlsbad, CA, USA) and fixed in 1.9% paraformaldehyde (Sigma-Aldrich). Cells were stored in FACS buffer (2 mM EDTA and 0.5% BSA in PBS) at 4°C until analyses.

### Isolation of immune cells from liver

Livers were dissected, washed in PBS and collected in RPMI 1640 GlutaMAX medium (Life Technologies, Grand Island, NY, USA). Immune cells from liver were isolated as described previously [19]. In brief, after mincing, tissues were digested for 20 minutes at 37°C in RPMI 1640 GlutaMAX (Life Technologies) supplemented with 1 mg/mL collagenase type IV from *C*. *hystolyticum* (Sigma-Aldrich), 2000 U/mL DNAse type I (Sigma-Aldrich) and 1 mM CaCl_2_ to activate the enzymes. Digestion was stopped by adding ice cold wash buffer (1% FCS and 2.5 mM EDTA in PBS) and digested tissues were filtered through a 100 μm cell strainer (Corning, Corning, NY, USA). Following pelleting cells twice at 1,500 rpm for 5 minutes at 4°C, hepatocytes were pelleted by spinning at 50x g for 3 minutes at 4°C. Supernatant was collected, centrifuged at 1,500 rpm for 5 minutes at 4°C, and pellet was treated with 5 mL red blood cell lysis buffer. Cells were manually counted, stained and fixed as described above.

### Flow cytometry

Stromal vascular cells and liver immune cells were stained for 30 min at 4°C in the dark with the fluorescently-labeled antibodies listed in S1 Table. To assess the macrophage M2-like phenotype, cells were first permeabilized with eBioscience permeabilization/wash buffer (San Diego, CA, USA) and stained with a biotin-conjugated Ym1 antibody (R&D systems, Minneapolis, MN, USA). All flow cytometry analyses were done within 3 days following cell fixation. Cells were measured by use of the FACSCanto flow cytometer (BD Bioscience, CA, USA) and analyzed using FlowJo software (Treestar, OR, USA). Representative gating schemes are shown in S1 Fig.

### Intraperitoneal glucose tolerance test

At 12 weeks LFD or HFD feeding, an intraperitoneal glucose tolerance test (ipGTT) was performed. Prior to the ipGTT, mice were fasted for 6 hours (from 8:00 am to 14:00 pm). Blood samples were collected by tail vein bleeding immediately at baseline (t = 0 min) and 5, 15, 30, 60, 90 and 120 minutes after intraperitoneal injection with glucose (2 g/kg body weight). Plasma glucose concentrations were quantified using the Glucose Start Reagent Method according to manufacturer’s instructions (Instruchemie, Delftzijl, The Netherlands).

### Plasma parameters

6 hour-fasted (from 8:00 am to 14:00 pm) blood samples were collected by tail vein bleeding into chilled capillaries and isolated plasma was assayed for glucose and insulin at week 0, 4, and 8. Glucose was measured using an enzymatic kit from Instruchemie (Delfzijl, the Netherlands), and insulin by ELISA (Crystal Chem Inc., Downers Grove, IL).

### RNA isolation and quantitative RT-PCR

RNA was extracted from snap-frozen mWAT and liver samples using the NucleoSpin RNA kit according to manufacturer’s instructions (Machery-Nagel, Düren, Germany). Concentrations and purity of RNA were determined on a NanoDrop ND-1000 spectrophotometer (Isogen, Maarssen, The Netherlands) and RNA was reverse transcribed using Moloney Murine Leukemia Virus Reverse Transcriptase (Promega, The Netherlands). Expression levels of genes were determined by qRT-PCR, using SYBR green supermix (Biorad, The Netherlands) and gene-specific primers (S2 Table). mRNA expression was normalized to cyclophilin (CypA) RNA and expressed as fold change versus control mice using the ΔΔCT method.

### Statistical analysis

Data are presented as means ± SEM. Statistical significance of differences was assessed by two-way ANOVA analysis of variance followed by a Tukey’s post hoc multiple comparison test to determine Interaction effect, HFD effect, and MOS effect. Body weight gain, fat mass gain, lean mass gain, cumulative food intake, plasma glucose, plasma insulin, and ipGTT were analyzed using two-way ANOVA for repeated measured, followed by a Tukey’s post hoc multiple comparison test. The results were considered statistically significant at *P*<0.05. Analyses were performed using Graph Pad Prism version 7.0 (GraphPad Software, San Diego, CA, USA).

## Results

### MOS supplementation did not affect body weight, fat mass, organ weight, and food intake

**Fig 1:**
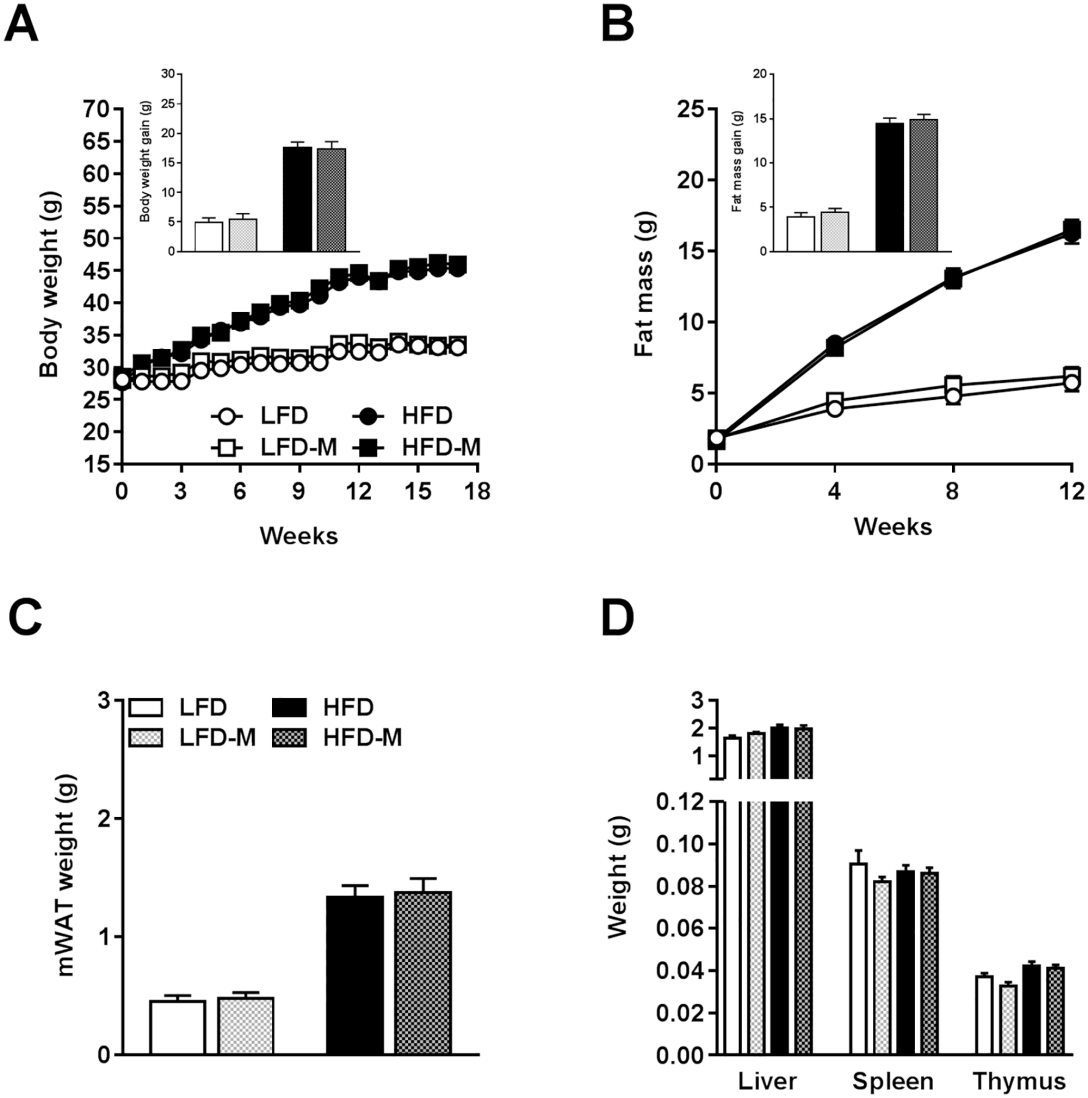
MOS supplementation did not affect body weight, fat mass, organ weight, and food intake. Body weight [A], fat mass [B], mWAT weight [C], organ weight of liver, spleen, and thymus weight [D] of mice fed a LFD or HFD with or without MOS for 17 weeks. Values are presented as means ± SEM (n = 10 mice/group). Differences were evaluated for statistical significance by two-way ANOVA or two-way ANOVA for repeated measurements, both followed by a Tukey’s post hoc multiple comparison test and provided in Table 1. mWAT, mesenteric white adipose tissue.

To assess the effect of MOS supplementation on diet-induced obesity, mice were fed a LFD or HFD supplemented with or without MOS for 17 weeks. As expected, HFD induced a time-dependent increase in body weight (*P*<0.0001; Fig 1A; Table 1), fat mass gain (*P*<0.0001; Fig 1B; Table 1), mWAT weight (*P*<0.0001; Fig 1C; Table 1), and lean mass gain (*P*<0.0001; Table 1; S2A Fig) when compared with LFD-fed mice. Furthermore, HFD significantly increased liver weight (*P* = 0.014; Fig 1D; Table 1) and thymus weight (*P* = 0.001; Fig 1D; Table 1). MOS supplementation did not affect body weight (Fig 1A; Table 1), fat mass (Fig 1B; Table 1), and lean mass (Table 1; S2A Fig) when compared to control diets. Accordingly, the weights of mWAT (Fig 1C; Table 1), liver, spleen, and thymus (Fig 1D; Table 1) were not affected by MOS supplementation. Finally, neither HFD feeding nor MOS supplementation affected cumulative food intake (Table 1; S2B Fig).

**Table 1:** Body weight, organ weight, and food intake characteristics.

Body weight and organ weight	LFD *(n = 10)*	LFD-M *(n = 10)*	HFD *(n = 10)*	HFD-M *(n = 10)*	Interaction effect	HFD effect	MOS effect	Time point effect
	*(mean ± SEM)*	*(mean ± SEM)*	*(mean ± SEM)*	*(mean ± SEM)*	*P-value*	*P-value*	*P-value*	*P-value*
Body weight gain (g)	4.99 ± 0.7165	5.48 ± 0.93	17.64 ± 0.89	17.39 ± 1.21	0.700	**<0.0001**	0.900	**<0.0001**
Fat mass gain (g)	3.88 ± 0.49	4.4 ± 0.47	14.45 ± 0.61	14.89 ± 0.59	0.945	**<0.0001**	0.387	**<0.0001**
mWAT weight (g)	0.45 ± 0.05	0.48 ± 0.05	1.33 ± 0.1	1.37 ± 0.12	0.944	**<0.0001**	0.706	n.a.
Liver weight (g)	1.64 ± 0.09	1.8 ± 0.06	2 ± 0.12	1.97 ± 0.13	0.354	**0.014**	0.523	n.a.
Spleen weight (g)	0.09 ± 0.01	0.08 ± 0.002	0.09 ± 0.003	0.09 ± 0.003	0.368	0.993	0.276	n.a.
Thymus weight (g)	0.04 ± 0.001	0.03 ± 0.002	0.04 ± 0.002	0.04 ± 0.002	0.348	**0.001**	0.140	n.a.
Lean mass gain (g)	1.26 ± 0.29	1.46 ± 0.38	2.63 ± 0.23	1.85 ± 0.35	0.129	**0.008**	0.370	**<0.0001**
Cumulative food intake week 17 (g / mouse)	52.7 ± 1.65	54.02 ± 1.70	49.96 ± 1.55	54.2 ± 2.77	0.472	0.527	0.179	**<0.0001**

*P<0*.*05* was considered significant determined by two-way ANOVA or two-way ANOVA for repeated measurements, both followed by a Tukey’s post hoc multiple comparison test;

Bold = (trend toward) significance;

mWAT = mesenteric white adipose tissue

### MOS supplementation reduced the abundance of M2-like monocytes and increased eosinophils in mWAT

The immune cell composition of WAT, specifically the balance between M1-like and M2-like macrophages and the abundance of eosinophils, has been shown to play a crucial role in the maintenance of adipocyte insulin sensitivity and whole-body metabolic homeostasis [20,21]. To assess whether MOS supplementation has extra-intestinal immune modulatory effects in WAT, the SVF was isolated from mWAT and the immune cell composition of the mWAT SVF was determined using flow cytometry (see S1 Fig for the gating scheme). The expression of CD11c and Ym1 within the total macrophage population allowed to discriminate between M1-like (YM1-CD11c+) and M2-like (YM1+CD11c-) macrophages, respectively [22].

**Fig 2:**
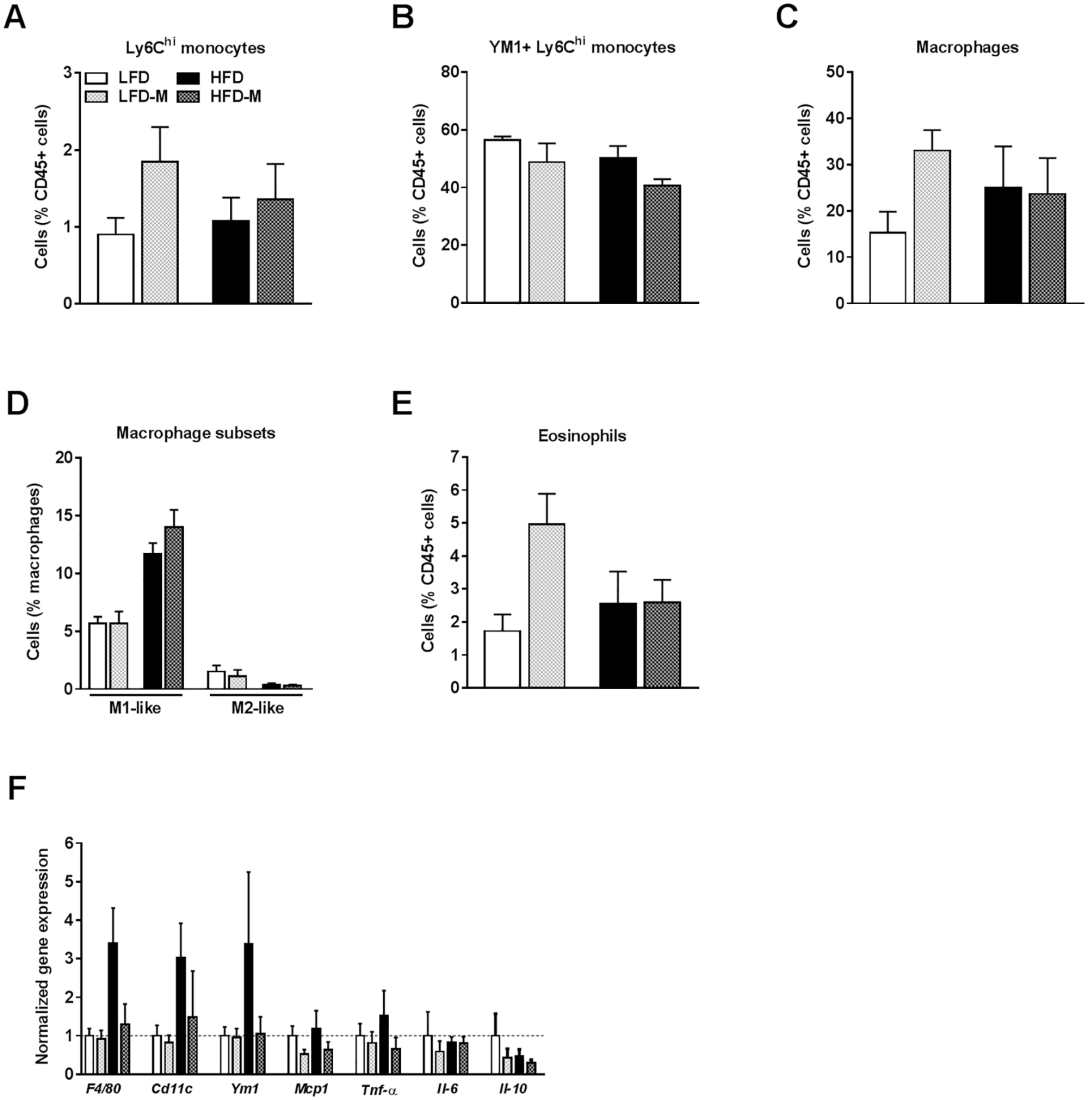
MOS supplementation reduced the abundance of M2-like monocytes and increased eosinophils in mWAT. Extra-intestinal immune modulatory properties of MOS were assessed in mWAT of mice fed a LFD or HFD with or without MOS for 17 weeks. Percentages of Ly6C^hi^ monocytes [A], YM1+ Ly6C^hi^ monocytes [B] macrophages [C], macrophage M1-like and M2-like subsets [D], and eosinophils [E] within CD45+ cells in SVF of mWAT. mRNA expression of the inflammatory markers *F4/80*, *CD11c*, *Ym1*, *Mcp1*, *Tnf-a*, *Il-6*, and *Il-10* was determined [F]. Values are presented as means ± SEM (n = 6–7 mice/group). Differences were evaluated for statistical significance by by two-way ANOVA, followed by a Tukey’s post hoc multiple comparison test and provided in Table 2. For information on the immunological cell markers used in flow cytometry analysis, see Method section and Table 2.

HFD feeding did not affect the total Ly6C^hi^ monocyte population in mWAT (Fig 2A; Table 2). However, a trend towards a diet effect (LFD/HFD) was observed for M1-like (CD11c+ Ly6C^hi^)(*P* = 0.052; Table 2) and M2-like (YM1+ LyC6^hi^) monocytes (*P* = 0.098; Fig 2B; Table 2). The total Ly6C^hi^ monocyte population in mWAT of MOS supplemented mice was not affected (Fig 2A; Table 2). However, mice that received MOS displayed a decrease in M2-like monocytes (*P* = 0.039; Fig 2B; Table 2). MOS did not affect M1-like monocytes (Table 2).

**Table 2:** Innate immune cells, lymphocytes, and relative gene expression characteristics in mWAT.

**Innate immune cells**	**Immunological cell markers**	**LFD** *(n = 7)*	**LFD-M** *(n = 6)*	**HFD** *(n = 5)*	**HFD-M** *(n = 6)*	**Interaction effect**	**HFD effect**	**MOS effect**
**mWAT**		*(mean ± SEM)*	*(mean ± SEM)*	*(mean ± SEM)*	*(mean ± SEM)*	*P-value*	*P-value*	*P-value*
Ly6C^hi^ monocytes (% CD45)	CD45+ Siglec-F- CD11b+ Ly6C^hi^ F4/80-	0.9 ± 0.2	1.85 ± 0.41	1.08 ± 0.27	1.36 ± 0.42	0.376	0.680	0.114
CD11c+ Ly6C^hi^ monocytes (%)	CD45+ Siglec-F- CD11b+ Ly6C^hi^ F4/80- CD11c+	50.6 ± 4.35	40 ± 6.47	35.6 ± 4.32	32.5 ± 4.18	0.503	**0.053**	0.224
YM1+ Ly6C^hi^ monocytes (%)	CD45+ Siglec-F- CD11b+ Ly6C^hi^ F4/80- YM1+	56.4 ± 1.16	48.8 ± 5.95	50.3 ± 3.65	40.7 ± 2.02	0.794	**0.085**	0.039*
Macrophages (% CD45)	CD45+ Siglec-F- Ly6C- CD11b+ F4/80+	15.2 ± 4.27	33 ± 4.07	25.1 ± 7.99	23.7 ± 7.1	0.151	0.973	0.217
M1-like macrophages (%)	CD45+ Siglec-F- Ly6C- CD11b+ F4/80+ YM1- CD11c+	5.67 ± 0.54	5.68 ± 1	11.7 ± 0.85	14 ± 1.4	0.295	<0.0001*	0.292
M2-like macrophages (%)	CD45+ Siglec-F- Ly6C- CD11b+ F4/80+ YM1+ CD11c-	1.51 ± 0.5	1.08 ± 0.53	0.34 ± 0.13	0.29 ± 0.09	0.667	0.038*	0.590
M2/M1 ratio		0.28 ± 0.10	0.26 ± 0.12	0.03 ± 0.01	0.02 ± 0.004	0.960	0.013*	0.853
Eosinophils (% CD45)	CD45+ Siglec-F+ (F4/80+)^$^	1.72 ± 0.46	4.95 ± 0.86	2.55 ± 0.88	2.59 ± 0.63	**0.052**	0.329	0.047*
Neutrophils (% CD45)	CD45+ Siglec-F- CD11b^hi^ Ly6C+ F4/80- YM1^hi^	0.52 ± 0.13	1.17 ± 0.49	1.05 ± 0.63	0.27 ± 0.07	**0.093**	0.655	0.879
**Lymphocytes**	**Immunological cell markers**	**LFD** *(n = 9)*	**LFD-M** *(n = 6)*	**HFD** *(n = 5)*	**HFD-M** *(n = 6)*			
**mWAT**		*(mean ± SEM)*	*(mean ± SEM)*	*(mean ± SEM)*	*(mean ± SEM)*	*P-value*	*P-value*	*P-value*
T cells (% CD45)	CD45+ NK1.1- CD3+	27.3 ± 3.44	18.8 ± 2.87	24.3 ± 4.03	17.1 ± 3.77	0.878	0.567	**0.062**
CD4+ T cells (%)	CD45+ NK1.1- CD3+ CD4+ CD8-	53.2 ± 2.59	46.2 ± 7.91	54.7 ± 1.62	30.8 ± 9.93	0.893	0.912	0.187
CD25+ CD4+ T cells (%)	CD45+ NK1.1- CD3+ CD4+ CD8- CD25+	1839 ± 49.5	1884 ± 92.1	1919 ± 83.9	2029 ± 90.3	0.709	0.197	0.369
CD8+ T cells (%)	CD45+ NK1.1- CD3+ CD8+ CD4-	29.7 ± 3.65	20.9 ± 2.87	31 ± 4.7	39.2 ± 8.42	0.153	0.102	0.951
CD25+ CD8+ T cells (%)	CD45+ NK1.1- CD3+ CD8+ CD4- CD25+	2084 ± 63	2069 ± 113.6	2002 ± 58.8	1857 ± 117.1	0.522	0.156	0.435
NK T cells (% CD45)	CD45+ NK1.1+ CD3+	6.5 ± 0.91	8.26 ± 0.86	6.86 ± 1.39	9.94 ± 2.03	0.654	0.492	0.110
NK cells (% CD45)	CD45+ NK1.1+ CD3-	4.2 ± 0.85	5.80 ± 0.72	2.91 ± 0.66	2.55 ± 0.35	0.248	0.011*	0.462
B cells (% CD45)	CD45+ CD19+ CD3- NK1.1-	34.9 ± 3.2	26.1 ± 4.82	36.3 ± 5.88	33.4 ± 5.77	0.576	0.413	0.277
**Gene expression**		**LFD** *(n = 8)*	**LFD-M** *(n = 7)*	**HFD** *(n = 7)*	**HFD-M** *(n = 4)*			
**mWAT**		*(mean ± SEM)*	*(mean ± SEM)*	*(mean ± SEM)*	*(mean ± SEM)*	*P-value*	*P-value*	*P-value*
F4/80		0.45 ± 0.09	0.46 ± 0.11	1.69 ± 0.46	0.64 ± 0.26	**0.086**	0.023*	**0.066**
CD11c		0.19 ± 0.05	0.15 ± 0.04	0.9 ± 0.36	0.27 ± 0.22	0.277	0.042*	0.177
Ym1		0.27 ± 0.06	0.26 ± 0.06	1.32 ± 0.56	0.29 ± 0.12	0.175	0.142	0.158
Mcp-1		0.68 ± 0.17	0.36 ± 0.08	0.81 ± 0.31	0.436 ± 0.13	0.902	0.634	0.120
Tnf-α		0.69 ± 0.22	0.56 ± 0.2	1.06 ± 0.44	0.46 ± 0.21	0.458	0.684	0.256
IL-6		0.3 ± 0.19	0.18 ± 0.08	0.25 ± 0.04	0.24 ± 0.05	0.662	0.965	0.629
IL-10		0.33 ± 0.19	0.14 ± 0.08	0.15 ± 0.06	0.1 ± 0.03	0.620	0.423	0.380

*P<0.*05* was considered significant determined by two-way ANOVA followed by a Tukey’s post hoc multiple comparison test;

^$^ Specific for mWAT

Bold = (trend toward) significance;

mWAT = mesenteric white adipose tissue

HFD feeding did not change the total abundance of macrophages (Fig 2C; Table 2). Although HFD did not affect the total abundance of macrophages in mWAT, HFD feeding induced a significant increase in M1-like macrophages (*P*<0.0001; Fig 2D; Table 2), a decrease in M2-like macrophages (*P* = 0.038; Fig 2D; Table 2), and a decreased M2/M1 ratio (*P* = 0.013; Table 2) in mWAT. MOS supplementation did not affect the total abundance of macrophages (Fig 2C; Table 2) and neither resulted in changes in M1-like and M2-like macrophage subsets (Fig 2D; Table 2), nor M2/M1 ratio (Table 2) in mWAT.

We further investigated whether granulocyte percentages within the CD45+ population of mWAT were affected by either HFD feeding or MOS supplementation. HFD did not significantly change eosinophils (Fig 2E; Table 2) or neutrophils (Table 2) in mWAT. MOS supplementation did not affect the neutrophil population (Table 2) in mWAT of both LFD- and HFD-fed mice. However, with respect to eosinophils in mWAT there was a tendency towards an interaction of MOS with diet (*P* = 0.052; Fig 2E; Table 2) as MOS doubled the percentage of eosinophils in LFD but not in HFD-fed mice (5.08% and 1.59% respectively, *P* = 0.047; Fig 2E; Table 2).

Finally, the effect of MOS supplementation on lymphocyte percentages within the CD45+ population was determined. HFD did not affect percentages of T cells, CD4+ T cells, CD8+ T cells, NK T cells, and B cells (Table 2), but lowered NK cells in (*P* = 0.011; Table 2). There were no effects of MOS supplementation on any of these cells, except for a trend toward decreased T cells (*P* = 0.062; Table 2).

Analysis of the mWAT mRNA gene expression showed that both the macrophage marker *F4/80* (*P* = 0.023; Fig 2F; Table 2) and the M1-like macrophage marker *CD11c* (*P* = 0.042; Fig 2F; Table 2) were increased in response to HFD. The relative mRNA expression of *CD11c*, *Ym1*, *Mcp1*, *Tnf-a*, *IL-6*, and *IL-10* was not affected by MOS supplementation (Fig 2F; Table 2). However, MOS showed a trend toward a decreased *F4/80* expression mainly on HFD (*P* = 0.066; Fig 2F; Table 2) which was likely due to an interaction with diet (LFD/HFD)(*P* = 0.086; Fig 2F; Table 2).

Taken together, these results showed that MOS supplementation has extra-intestinal immune modulatory properties by reducing M2-like monocytes on both diets and increasing eosinophils on LFD, whilst showing a trend toward reduced T cells on both diets and *F4/80* expression on HFD in mWAT.

### MOS supplementation slightly affected hepatic monocytes and macrophage subsets

Classical activation of Kupffer cells, the liver-resident macrophages, has been observed in diet-induced obesity [23]. Therefore, we determined the effect of MOS supplementation on hepatic immune cell composition using flow cytometry (see S1 Fig for the gating scheme).

**Fig 3:**
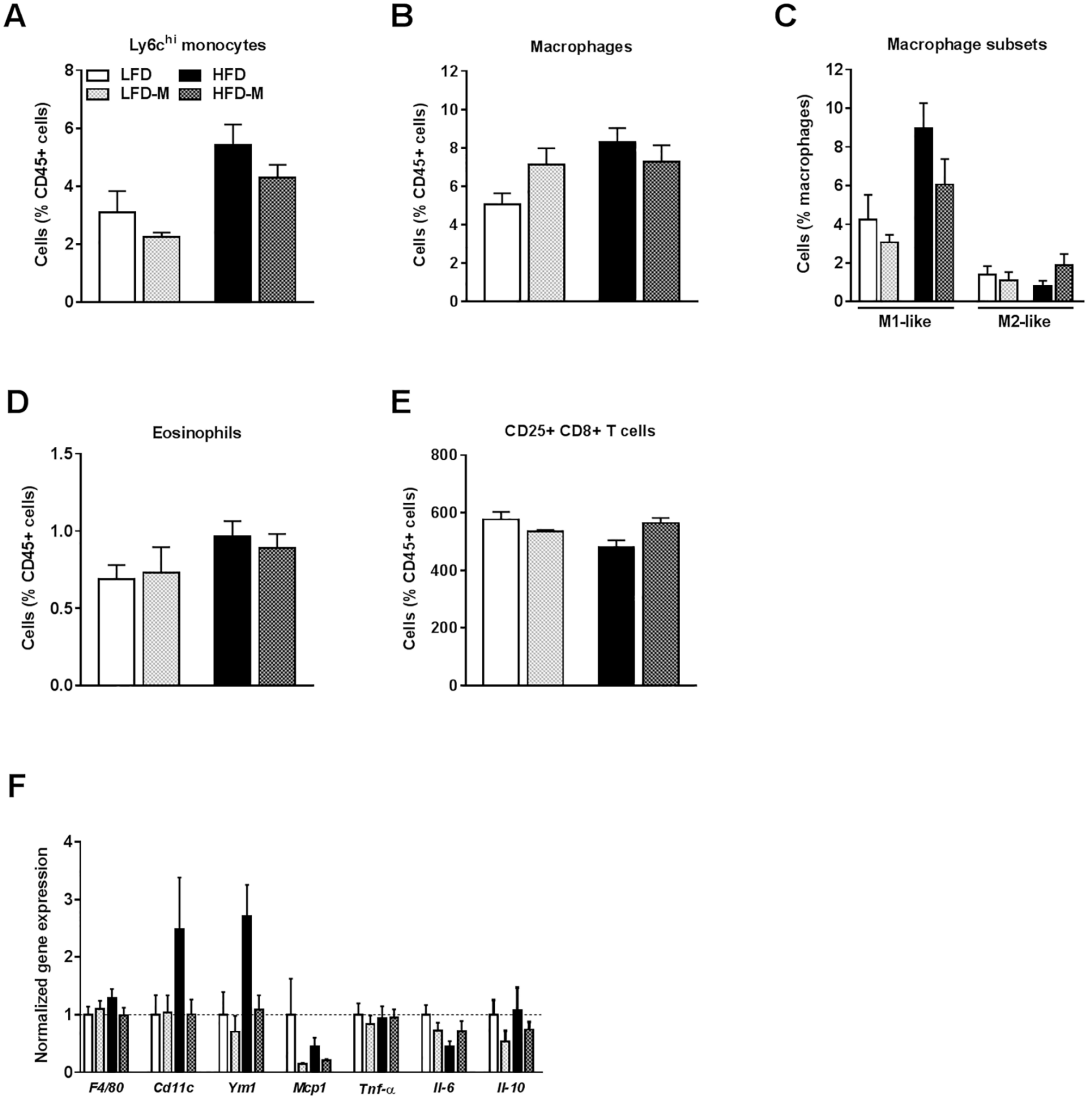
MOS supplementation slightly affected hepatic monocytes and macrophage subsets. Hepatic extra-intestinal immune modulatory properties of MOS were assessed in mice fed a LFD or HFD with or without MOS for 17 weeks. Percentages of Ly6C^hi^ monocytes [A], macrophages [B], macrophage M1-like and M2-like subsets [C], eosinophils [D] and CD25+ CD8+ expressing T cells [E] within CD45+ cells in the liver. mRNA expression of the inflammatory markers *F4/80*, *CD11c*, *Ym1*, *Mcp1*, *Tnf-a*, *Il-6*, and *Il-10* was determined [F]. Values are presented as means ± SEM (n = 4–10 mice/group). Differences were evaluated for statistical significance by by two-way ANOVA, followed by a Tukey’s post hoc multiple comparison test and provided in Table 3. For information on the immunological cell markers used in flow cytometry analysis, see Method section and Table 3.

As expected, HFD feeding increased Ly6C^hi^ monocytes (*P* = 0.001; Fig 3A; Table 3) and macrophages (*P* = 0.032; Fig 3B; Table 3) in the liver, indicating enhanced recruitment of pro-inflammatory monocytes. After MOS supplementation, a trend towards decreased Ly6C^hi^ monocytes were observed (*P* = 0.093; Fig 3A; Table 3). Accordingly, an interaction was found between MOS supplementation and diet (LFD/HFD) on the total percentage of macrophages in the liver (*P* = 0.05; Fig 3B; Table 3).

**Table 3:** Innate immune cells, lymphocytes, and relative gene expression characteristics in liver.

**Innate immune cells**	**Immunological cell markers**	**LFD** *(n = 10)*	**LFD-M** *(n = 9)*	**HFD** *(n = 10)*	**HFD-M** *(n = 10)*	**Interaction effect**	**HFD effect**	**MOS effect**
**Liver**		*(mean ± SEM)*	*(mean ± SEM)*	*(mean ± SEM)*	*(mean ± SEM)*	*P-value*	*P-value*	*P-value*
Ly6C^hi^ monocytes (% CD45)	CD45+ Siglec-F- CD11b+ Ly6C^hi^ F4/80-	3.10 ± 0.69	2.24 ± 0.15	5.42 ± 0.67	4.29 ± 0.43	0.812	0.001*	**0.093**
CD11c+ Ly6C^hi^ monocytes (%)	CD45+ Siglec-F- CD11b+ Ly6C^hi^ F4/80- CD11c+	9.52 ± 1.57	8.17 ± 0.76	8.88 ± 0.77	10.9 ± 1.14	0.164	0.384	0.778
YM1+ Ly6C^hi^ monocytes (%)	CD45+ Siglec-F- CD11b+ Ly6C^hi^ F4/80- YM1+	20.6 ± 4.48	13.1 ± 2.79	15.8 ± 5	27 ± 6.24	**0.078**	0.378	0.724
Macrophages (% CD45)	CD45+ Siglec-F- Ly6C- CD11b+ F4/80+	5.06 ± 0.54	7.12 ± 0.82	8.30 ± 0.69	7.27 ± 0.81	0.050*	0.032*	0.502
M1-like macrophages (%)	CD45+ Siglec-F- Ly6C- CD11b+ F4/80+ YM1- CD11c+	4.23 ± 1.23	3.06 ± 0.34	8.94 ± 1.25	6.05 ± 1.26	0.469	0.003*	**0.095**
M2-like macrophages (%)	CD45+ Siglec-F- Ly6C- CD11b+ F4/80+ YM1+ CD11c-	1.38 ± 0.43	1.1 ± 0.4	0.80 ± 0.26	1.9 ± 0.54	0.131	0.809	0.366
M2/M1 ratio		0.68 ± 0.3	0.45 ± 0.2	0.13 ± 0.06	0.62 ± 0.2	0.108	0.398	0.552
Eosinophils (% CD45)	CD45+ Siglec-F+ (F4/80+)	0.69 ± 0.09	0.73 ± 0.15	0.97 ± 0.09	0.89 ± 0.09	0.599	**0.061**	0.888
Neutrophils (% CD45)	CD45+ Siglec-F- CD11b^hi^ Ly6C+ F4/80- YM1^hi^	6.20 ± 1.44	7.01 ± 1.48	5.04 ± 1.41	6.23 ± 1.33	0.875	0.539	0.491
**Lymphocytes**	**Immunological cell markers**	**LFD** *(n = 6)*	**LFD-M** *(n = 4)*	**HFD** *(n = 5)*	**HFD-M** *(n = 6)*			
**Liver**		*(mean ± SEM)*	*(mean ± SEM)*	*(mean ± SEM)*	*(mean ± SEM)*	*P-value*	*P-value*	*P-value*
T cells (% CD45)	CD45+ NK1.1- CD3+	10.8 ± 0.79	9.29 ± 0.42	11.9 ± 1.45	10.2 ± 0.86	0.915	0.369	0.172
CD4+ T cells (%)	CD45+ NK1.1- CD3+ CD4+ CD8-	54.1 ± 1.43	55.6 ± 1.27	56.4 ± 6.04	56.1 ± 0.99	0.800	0.698	0.866
CD25+ CD4+ T cells (%)	CD45+ NK1.1- CD3+ CD4+ CD8- CD25+	367.8 ± 14.11	363.8 ± 21.85	338.2 ± 18.87	364 ± 6.95	0.395	0.403	0.534
CD8+ T cells (%)	CD45+ NK1.1- CD3+ CD8+ CD4-	37.6 ± 1.76	34.1 ± 2.91	34.8 ± 5.83	34.8 ± 0.83	0.635	0.769	0.624
CD25+ CD8+ T cells (%)	CD45+ NK1.1- CD3+ CD8+ CD4- CD25+	576 ± 24.6	534.3 ± 5.14	480 ± 21.88	563.5 ± 16.95	0.013*	0.159	0.370
NK T cells (% CD45)	CD45+ NK1.1+ CD3+	10.4 ± 0.83	10.2 ± 0.62	7.56 ± 1.29	10.7 ± 0.76	0.126	0.265	0.189
NK cells (% CD45)	CD45+ NK1.1+ CD3-	3.46 ± 0.27	3.88 ± 0.67	3.32 ± 0.5	3.56 ± 0.25	0.853	0.623	0.482
B cells (% CD45)	CD45+ CD19+ CD3- NK1.1-	35.6 ± 1.41	34.1 ± 0.53	23 ± 4.98	28.3 ± 1.06	0.268	0.006*	0.523
**Gene expression**		**LFD** *(n = 10)*	**LFD-M** *(n = 9)*	**HFD** *(n = 9)*	**HFD-M** *(n = 10)*			
**Liver**		*(mean ± SEM)*	*(mean ± SEM)*	*(mean ± SEM)*	*(mean ± SEM)*	*P-value*	*P-value*	*P-value*
F4/80		0.73 ± 0.11	0.81 ± 0.1	0.94 ± 0.12	0.73 ± 0.1	0.176	0.536	0.494
CD11c		0.25 ± 0.08	0.26 ± 0.08	1 ± 0.43	0.25 ± 0.06	**0.092**	**0.098**	**0.099**
Ym1		0.41 ± 0.16	0.29 ± 0.11	1.11 ± 0.23	0.45 ± 0.1	0.103	0.012*	0.021*
Mcp-1		0.44 ± 0.28	0.06 ± 0.009	0.2 ± 0.07	0.09 ± 0.01	0.374	0.478	0.113
Tnf-α		0.452 ± 0.09	0.38 ± 0.07	0.42 ± 0.1	0.43 ± 0.07	0.620	0.898	0.684
IL-6		1.18 ± 0.12	0.85 ± 0.17	0.53 ± 0.11	0.84 ± 0.21	**0.080**	**0.072**	0.967
IL-10		0.78 ± 0.18	0.37 ± 0.13	0.75 ± 0.28	0.52 ± 0.09	0.797	0.583	0.130

*P<0.*05* was considered significant determined by two-way ANOVA followed by a Tukey’s post hoc multiple comparison test;

Bold = (trend toward) significance

HFD-feeding increased predominantly M1-like macrophage subsets (*P* = 0.003; Fig 3C; Table 3), while MOS supplementation resulted in a tendency toward decreased M1-like macrophages (*P* = 0.095; Fig 3C; Table 3). No effects were found on M2-like macrophages (Fig 3C; Table 3) and on the M2/M1 ratio (Table 3) either with HFD or MOS.

We further investigated whether MOS supplementation affected granulocyte percentages within the CD45+ population of the liver. A tendency toward increased eosinophils was found after HFD feeding (*P* = 0.061; Fig 3D; Table 3), while neutrophils remained unaffected (Table 3). However, MOS-supplementation did not affect hepatic neutrophils (Table 3) or eosinophils (Fig 3D; Table 3).

Finally, we determined the effect of MOS on lymphocyte percentages within the CD45+ population. HFD did not affect percentages of total T cells, CD4+ T cells, CD8+ T cells, NK T cells, and NK cells (Table 3). However, B cells were found to be significantly lower in HFD-fed mice (*P* = 0.006; Table 3). MOS did not affect any of these lymphocytes, although a significant interaction was found between diet (LFD/HFD) and MOS on CD25+ CD8+ expressing T cells (*P* = 0.013; Fig 3E; Table 3).

Analysis of the liver mRNA gene expression showed an increase in the expression of *Ym1* (*P* = 0.012; Fig 3F; Table 3) in HFD-fed mice indicating M2-like macrophages, and we found a tendency towards an increase in *CD11c (P* = 0.098; Fig 3F; Table 3) indicating M1-like macrophages in HFD-fed mice. On the other hand, MOS decreased hepatic expression of *Ym1* (*P* = 0.021; Fig 3F; Table 3) and a tendency towards decreased expression of *CD11c* (*P* = 0.099; Fig 3F; Table 3). A trend towards interaction between diet (LFD/HFD) and MOS was found for the expression of CD11c (*P* = 0.092; Fig 3C; Table 3). Finally, HFD tended to decrease the expression of IL-6 (*P* = 0.072; Fig 3F; Table 3), and an interaction between diet (LFD/HFD) and MOS supplementation was found for IL-6 (*P* = 0.08; Fig 3F; Table 3). Gene expression of *F4/80*, *Mcp1*, *Tnf-a*, and *Il-10* remained unaffected by diet or MOS (Fig 3F; Table 3).

Overall, MOS supplementation modestly affected the liver with tendencies to decrease Ly6C^hi^ monocytes and M1-like macrophages.

### MOS supplementation did not improve whole-body glucose intolerance

**Fig 4:**
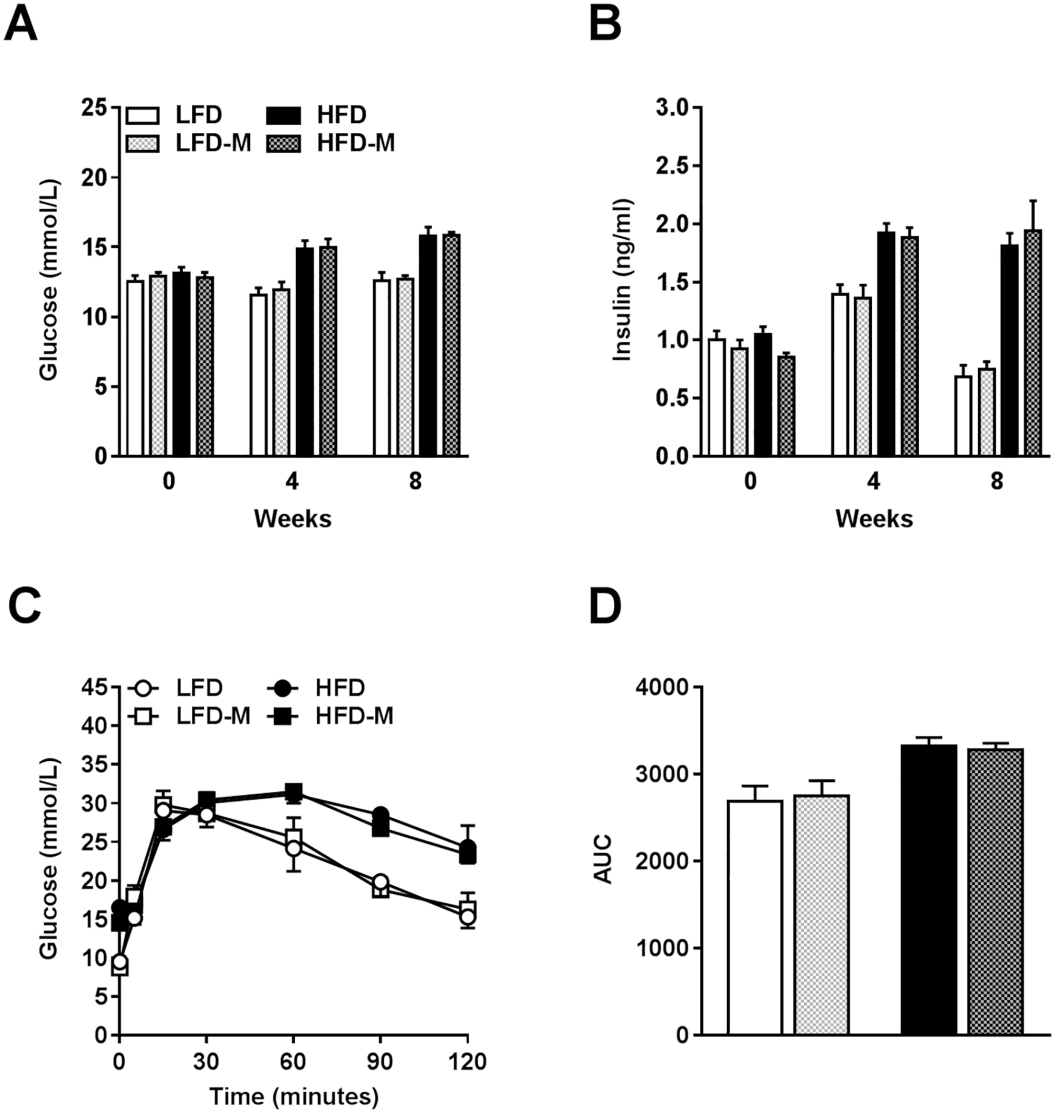
MOS supplementation did not improve whole-body glucose intolerance. Whole-body glucose homeostasis was assessed in mice fed a LFD or HFD with or without MOS for 17 weeks. Fasting plasma glucose [A] and insulin levels [B] were determined in 6-hour fasted mice in week 0, 4, and 8. An ipGTT was performed in 6-hour fasted mice at week 12. Blood glucose levels were measured at the indicated minutes [C], and the area under the curve (AUC) of the glucose excursion curve was calculated as a measure for glucose tolerance [D]. Values are presented as means ± SEM (n = 10 mice/group). Differences were evaluated for statistical significance by by two-way ANOVA or two-way ANOVA for repeated measurements, both followed by a Tukey’s post hoc multiple comparison test and provided in Table 4.

We next studied whether MOS affected whole-body glucose homeostasis in lean and diet-induced obese mice. As expected, HFD feeding increased fasting plasma glucose (*P*<0.0001; Fig 4A; Table 4) and insulin (*P*<0.0001; Fig 4B; Table 4) levels over time as compared to LFD feeding. In week 12, whole-body glucose tolerance was measured using ipGTT. HFD deteriorated glucose tolerance over time as compared to LFD-fed mice (*P*<0.0001; Fig 4C and 4D; Table 4). MOS supplementation did neither affect fasting plasma glucose (Fig 4A; Table 4) or insulin (Fig 4B; Table 4) levels, nor altered glucose tolerance (Fig 4C and 4D; Table 4). These data indicate that MOS supplementation did not affect whole-body glucose homeostasis.

**Table 4:** Glucose, insulin, and ipGTT characteristics.

Glucose, insulin, and ipGTT analysis	LFD *(n = 10)*	LFD-M *(n = 10)*	HFD *(n = 10)*	HFD-M *(n = 10)*	Interaction effect	HFD effect	MOS effect	Time point effect
	*(mean ± SEM)*	*(mean ± SEM)*	*(mean ± SEM)*	*(mean ± SEM)*	*P-value*	*P-value*	*P-value*	*P-value*
Glucose (mmol/L)	12.6 ± 0.58	12.71 ± 0.25	15.82 ± 0.62	15.85 ± 0.20	0.930	<0.0001*	0.876	<0.0001
Insulin (ng/ml)	0.68 ± 0.1	0.751 ± 0.06	1.81 ± 0.11	1.94 ± 0.26	0.850	<0.0001*	0.525	<0.0001
ipGTT (AUC)	2687 ± 175.1	2745 ± 179.3	3327 ± 91.7	3281 ± 73.7	0.710	0.0001*	0.968	<0.0001

*P<0.*05* was considered significant determined by two-way ANOVA or two-way ANOVA for repeated measurements followed, both by a Tukey’s post hoc multiple comparison test;

Bold = (trend toward) significance;

AUC = area under curve; ipGTT = intraperitoneal glucose tolerance test

## Discussion

The immune modulatory properties of MOS have been exploited to increase the economic yields of livestock [12,13]. In the present study, we investigated the effect of MOS supplementation on body weight and composition, food intake, immune composition of mWAT and liver, and whole-body glucose tolerance in both LFD-fed lean and HFD-induced obese mice. We showed that MOS supplementation mildly altered immune cell composition in both mWAT and liver, which was not accompanied by ameliorations in HFD-induced obesity or whole-body glucose intolerance. Our data confirm the potential extra-intestinal modulatory properties of MOS on immune composition as reported previously [9,14–16], although the effects are relatively modest.

Specifically, MOS increased eosinophils in mWAT of LFD-fed mice. Eosinophils have been shown previously to beneficially reduce inflammation in WAT by promoting M2-like macrophage polarization [5,20]. However, the observed increase in eosinophils with MOS did not lead to skewing toward M2-like macrophages as there were no alterations in macrophage subpopulations after MOS supplementation in mWAT. As a matter of fact, MOS even led to a decrease in M2-like monocytes in mWAT. The effects of MOS on eosinophils were therefore probably too small to induce beneficial effects on macrophage polarization. Whether MOS is able to induce more substantial effects in other fat depot regions remains to be investigated. As whole-body glucose tolerance was not affected by MOS supplementation in both lean and obese mice in our study, we suggest that MOS will not affect inflammation in any of the fat depots.

In livers of these mice, HFD feeding increased both the total amount of monocytes and macrophages. However, no significant effect of MOS was found on reducing these parameters although MOS tended to reduce monocytes and M1-like macrophages in the liver. This may imply that MOS supplementation in this setting steers towards beneficial M2/M1 ratios in the liver, although the observed effects were minor. Additionally, the data obtained from the flow cytometry data were not in direct line with the gene expression data. Although we do not have a clear explanation for this, it is likely that this discrepancy is a result of the markers measured on specific cell types on protein level in flow cytometry analysis versus markers measured on the total pool of cells on gene expression level.

Given that MOS supplementation did not affect diet-induced obesity and whole-body glucose tolerance, leads us to speculate that the observed alterations in immune cell compositions were insufficient to achieve a significant effect. Another possibility is that the concentration of supplemented MOS in the diet was not high enough. However, in previous studies where MOS supplementation showed intra-intestinal and extra-intestinal effects on the immune system, concentrations of 0.005% to 0.5% of MOS were used in the diet [10,14,24]. However, these studies were performed in broiler chickens or pigs, and it is possible that different species respond differently to MOS. Another study performed in mice also used 1% MOS supplementation and found a decreased fat accumulation in the parametrial adipose tissue and in the liver [17]. However, this latter study MOS derived from coffee mannan which is different from the yeast-derived MOS that we used in this study. Whether the origin of MOS may determine the effect of MOS on fat accumulation remains to be determined. The limited effects of MOS supplementation on diet-induced obesity in our mice did not seem to be due to inappropriate dosages of MOS.

Alternatively, other factors within the experimental setting might explain the relatively limited effects of MOS in our experiments. In previous studies, MOS showed anti-inflammatory effects in experimentally viral or bacterial infected animals [9,14–16]. In order for MOS to reduce inflammation, a strong pro-inflammatory trigger may first be needed, e.g. by bacteria or bacterial components such as LPS. Importantly, the experimental mice used in our study are guaranteed free of particular pathogens. The mechanistic action of MOS to improve performance in animal industry is thought to occur via the ability of MOS to inhibit attachment of pathogens with type-1 fimbriae to the intestinal wall of animals [25]. In our facility, the presence of type-1 fimbriae containing pathogenic bacteria residing in the gut of the mice is probably very limited. Further research needs to be conducted to determine whether *Saccharomyces cerevisiae*-derived MOS is dependent upon pathogenic stimuli in order to exert its anti-inflammatory function.

Furthermore, the impact of the HFD might be too strong in order for MOS to exert its beneficial function on the intestinal barrier. Intestinal epithelial mucosal surfaces possess a variety of defense mechanisms to prevent adhesion of bacteria, including mucus secretion and sloughing [26,27]. Mucins are major anti-adhesive components of mucus. In order for the epithelial surface to produce mucus, an intact epithelial layer should be present. HFD feeding in mice damages the intestinal barrier integrity, increasing intestinal permeability and increasing LPS leakage (endotoxemia) into the system [28]. It is likely that MOS is not able to restore the intestinal barrier integrity to inhibit bacterial colonization and reduce systemic inflammation.

The type of MOS used in various studies might also determine the effect of MOS on diet-induced obesity, glucose tolerance, and immune modulation. In our study, we used mannan derived from the yeast *saccharomyces cerevisiae*. However, MOS can be derived from various sources with different effects on body weight in mice. For instance, MOS derived from coffee mannan decreased fat accumulation in mice [17], whereas MOS derived from the plant konjac mannan did not have any effect on body weight in mice [29]. Therefore, it remains to be investigated whether MOS derived from different sources also have different immune modulatory effects.

In conclusion, this study showed that MOS supplementation did alter immune composition in mWAT and liver. However, these effects were not accompanied by ameliorations in HFD-induced glucose intolerance or inflammation.

## Supporting information

S1 Table Antibodies used for flow cytometry. (PDF)

S2 Table Primer sequences of forward and reverse primers (5’→3’). (PDF)

S1 Fig Gating strategies mWAT and liver. Isolated cells were pre-gated on Aqua-CD45+ single cells. FSC-A, forward scatter area; SSC-A, sideward scatter area; FSC-W, forward scatter width [A]. Gating strategies for the analysis of eosinophils, neutrophils and monocytes [B], macrophages, M1-like (CD11c+Ym1-) macrophages and M2-like (CD11c-Ym1+) macrophages [C], and NK cell, NK T cell, T cell and B cell lymphocyte subsets [D] are given. Gating strategies are shown for representative samples from mWAT and liver.
(PDF)

S2 Fig The effect of MOS supplementation on lean mass and cumulative food intake. Lean mass [A] and food intake [B] of mice fed a LFD or HFD with or without MOS for 17 weeks. Values are presented as means ± SEM (n = 10 mice/group). Differences were evaluated for statistical significance by two-way ANOVA for repeated measures, followed by Tukey’s post hoc multiple comparison test and provided in Table 1.
(TIF)
