# Supplementary material for: Dietary yeast-derived mannan oligosaccharides have immune-modulatory properties but do not improve high fat diet-induced obesity and glucose intolerance
Source: PLoS One. 2018 May 3;13(5):e0196165. doi: 10.1371/journal.pone.0196165 (PMC5933760; doi:10.1371/journal.pone.0196165)
Supplement: S1 Table — (PDF) [file pone.0196165.s001.pdf]

S1 Table. Antibodies used for flow cytometry

Fluorophore	Antibody	Clone	Vendor
FITC	anti-CD45.2	104	Biolegend
PE	anti-Siglec-F	E50-2440	BD Biosciences
PE	anti-NK1.1	PK136	BD Biosciences
PerCP	Streptavidin	N/A	BD Biosciences
PerCP-Cy5.5	anti-CD25	PC61	BD Biosciences
PE-Cy7	anti-CD11b	M1/70	eBioscience
PE-Cy7	anti-CD4	GK1.5	eBioscience
APC	anti-F4/80	BM8	eBioscience
APC	anti-CD8a	53-6.7	Biolegend
APC-Cy7	anti-Ly6C	HK1.4	Biolegend
APC-Cy7	anti-CD19	1D3	eBioscience
Horizon V450	anti-CD11c	HL3	BD Biosciences
eFluor 450	anti-CD3	17A2	eBioscience
