# Supplementary material for: Dietary yeast-derived mannan oligosaccharides have immune-modulatory properties but do not improve high fat diet-induced obesity and glucose intolerance
Source: PLoS One. 2018 May 3;13(5):e0196165. doi: 10.1371/journal.pone.0196165 (PMC5933760; doi:10.1371/journal.pone.0196165)
Supplement: S2 Table — (PDF) [file pone.0196165.s002.pdf]

S2 Table. Primer sequences of forward and reverse primers (5'→3').

Gene ID	Alias	Sense	Antisense
Ppia	*CypA*	ACTGAATGGCTGGATGGCAA	TGTCCACAGTCGGAAATGGT
Emr1	*F4/80*	CTTTGGCTATGGGCTTCCAGTC	GCAAGGAGGACAGAGTTTATCGTG
Itgax	*Cd11c*	*GCCACCAACCCTTCCTGGCTG*	*TTGGACACTCCTGCTGTGCAGTTG*
Chil3	*Ym1*	ACAATTAGTACTGGCCCACCAGGAA	TCCTTGAGCCACTGAGCCTTCA
Ccl2	*Mcp1*	CACTCACCTGCTGCTACTCA	GCTTGGTGACAAAAACTACAGC
Tnf	*Tnf-α*	GATCGGTCCCCAAAGGGATG	CACTTGGTGGTTTGCTACGAC
Il6	*Il6*	AAGAAATGATGGATGCTACCAAACTG	GTACTCCAGAAGACCAGAGGAAATT
Il10	*Il10*	GACAACATACTGCTAACCGACTC	ATCACTCTTCACCTGCTCCACT
