# Supplementary figures and images for: Dietary yeast-derived mannan oligosaccharides have immune-modulatory properties but do not improve high fat diet-induced obesity and glucose intolerance
Source: PLoS One. 2018 May 3;13(5):e0196165. doi: 10.1371/journal.pone.0196165 (PMC5933760; doi:10.1371/journal.pone.0196165)

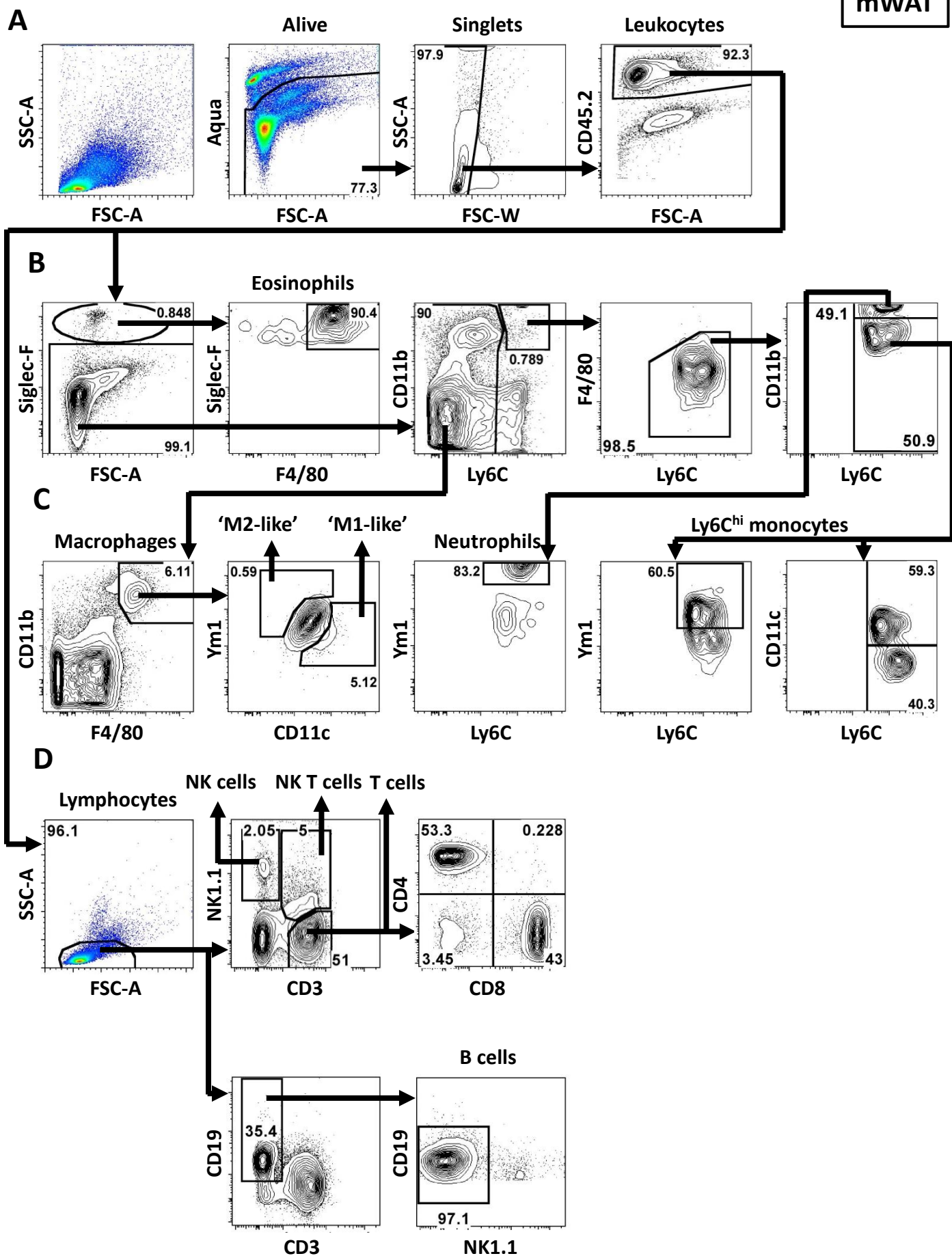

S1 Figure. Gating strategies mWAT and liver

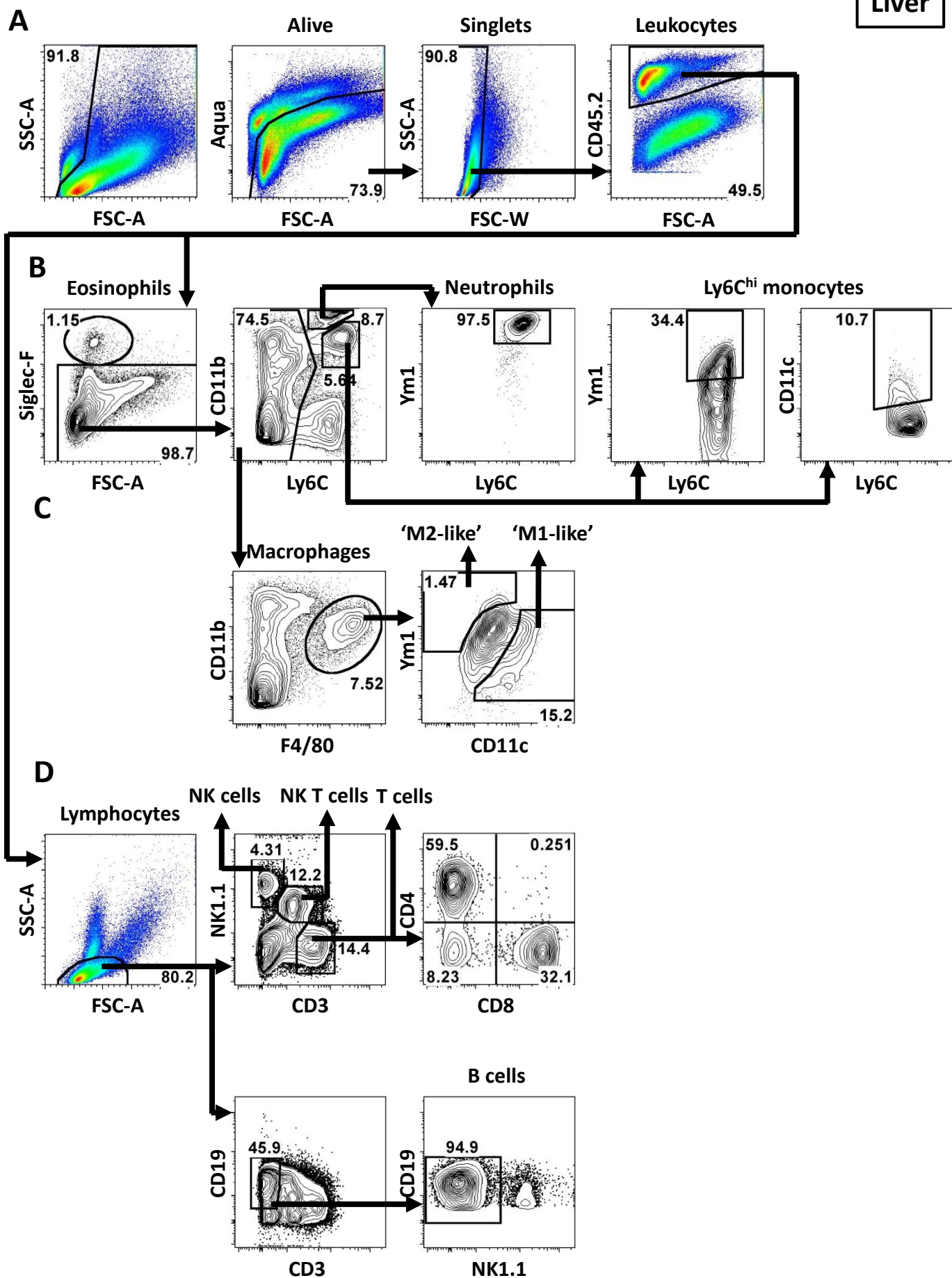

S1 Figure. Gating strategies mWAT and liver

Supplement: S1 Fig — Isolated cells were pre-gated on Aqua-CD45+ single cells. FSC-A, forward scatter area; SSC-A, sideward scatter area; FSC-W, forward scatter width [A]. Gating strategies for the analysis of eosinophils, neutrophils and monocytes [B], macrophages, M1-like (CD11c+Ym1-) macrophages and M2-like (CD11c-Ym1+) macrophages [C], and NK cell, NK T cell, T cell and B cell lymphocyte subsets [D] are given. Gating strategies are shown for representative samples from mWAT and liver. (PDF) [file pone.0196165.s003.pdf]

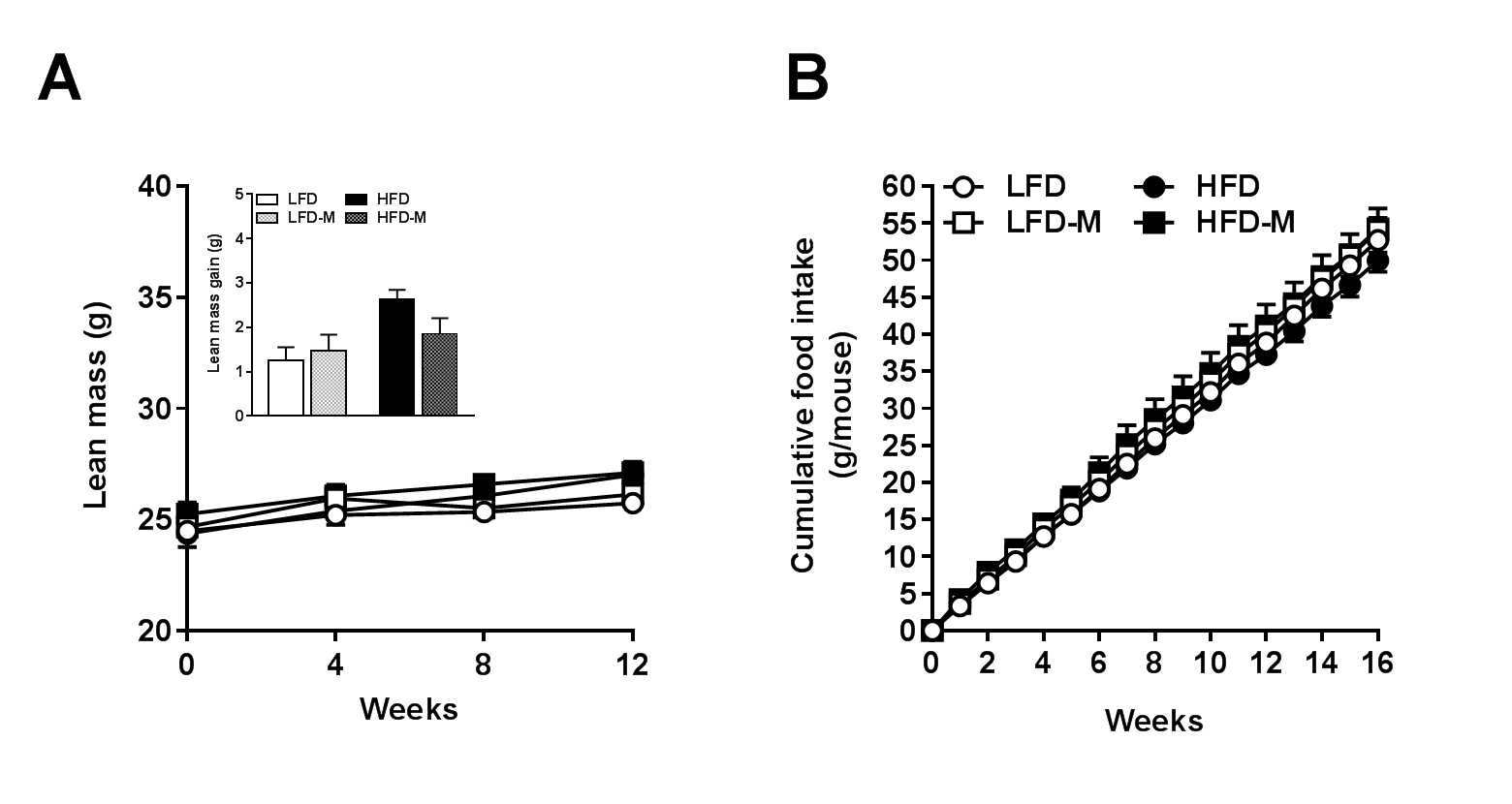

Supplement: S2 Fig — Lean mass [A] and food intake [B] of mice fed a LFD or HFD with or without MOS for 17 weeks. Values are presented as means ± SEM (n = 10 mice/group). Differences were evaluated for statistical significance by two-way ANOVA for repeated measures, followed by Tukey’s post hoc multiple comparison test and provided in Table 1. (TIF) [file pone.0196165.s004.tif]
